# Machine learning can classify vital sign alerts as real or artifact in online continuous monitoring data

**DOI:** 10.1186/2197-425X-3-S1-A550

**Published:** 2015-10-01

**Authors:** M Hravnak, L Chen, A Dubrawski, D Wang, E Bose, G Clermont, AM Kaynar, D Wallace, A Holder, MR Pinsky

**Affiliations:** School of Nursing, University of Pittsburgh, Pittsburgh, PA USA; Robotics Institute, Carnegie Mellon University, Pittsburgh, PA USA; School of Medicine, University of Pittsburgh, Pittsburgh, PA USA

## Introduction

Alarm hazards continue to be the top patient safety concern of 2015. Machine learning (ML) can be used to classify patterns in monitoring data to differentiate real alerts from artifact.

## Objectives

To determine the degree to which ML, specifically random forest (RF), can classify vital sign (VS) alerts in continuous monitoring data as they unfold online as either real alerts or artifact.

## Methods

Noninvasive monitoring data from 8 weeks of admissions in a 24-bed step-down unit (heart rate [HR], respiratory rate (RR; bioimpedance), oscillometric blood pressure (BP), peripheral oximetry (SpO_2_)) were recorded at 1/20Hz. VS deviation beyond stability thresholds (HR 40-140, RR 8-36, systolic BP 80-200, diastolic BP < 110, SpO_2_>85%) and persisting for 80% of a 5 min moving window comprised alerts. Of 1,582 alerts, 631 were labeled by a 4-member expert committee as real alerts, artifact, or unable to classify. Alerts were: RR 132 real, 25 artifact; BP 45 real, 40 artifact; SpO_2_ 181 real, 93 artifact (HR alerts too few to analyze). Following feature extraction from expert-annotated alerts, we constructed a series of 10 moving windows of 3 min width each, and ending at 0, 20, 40, 60, 80, 100, 120, 140, 160, and 180s from the time the VS first crossed alert threshold. The experiment is performed within a leave-one-alert-out setup. In each iteration, one of the alerts is the test alert, and the rest are used as the training alerts. We trained the model using only the windows ending at 180s after the time VS crossed the alert threshold from the training alerts (one for each VS), and then made predictions from each of the sliding windows on the test alert. We then computed area under the curve (AUC) scores by aggregating prediction at each test window.

## Results

The RF classifier was able to discriminate between real BP alerts and artifact using information from the prior 3 min with an AUC of 0.8 in the 0s window, which improved to 0.86 for the window ending at 180s into the alert. SpO2 has an AUC of 0.88 for the 0s window, and improved to 0.96 at 180s window. RR discrimination has an AUC of 0.73 at the 0s window, and improved to 0.92 at the 180s window.

## Conclusions

A RF model trained on a small set of expert-annotated data was able to accurately classify RR, BP and SpO_2_ alerts in monitored data as they are unfolding online as real or artifact to a helpful degree. BP and SpO_2_ did not improve much with more information gained after alert onset, while information gained as the alert continued to unfold improved RR discrimination. This approach holds promise to improve monitor alerting technology and clinical care.

## Grant Acknowledgment

NIH NINR R01NR013912; NSF 1320347; NHLBI-K08-HL122478

**Figure 1:**
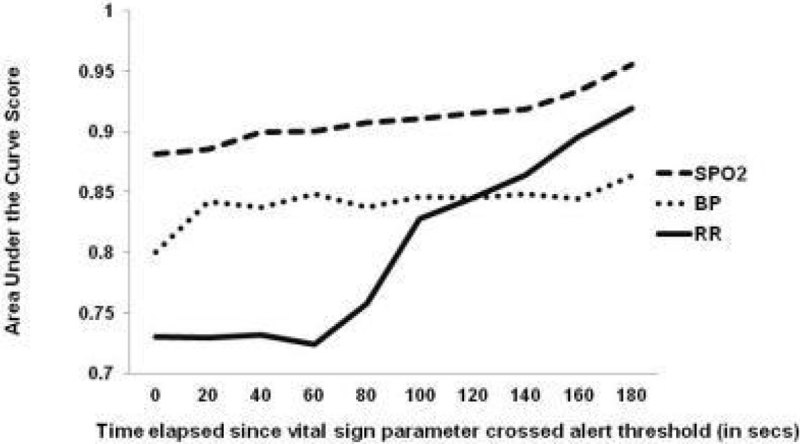
***Random forest Area Under the Curve scores for accurate discrimination of real alerts vs. artifact in vital sign abnormalities developing in online continuous monitoring data.***

